# N6-Methyladenosine Promotes the Transcription of c-Src Kinase via IRF1 to Facilitate the Proliferation of Liver Cancer

**DOI:** 10.32604/or.2025.062747

**Published:** 2025-06-26

**Authors:** Yanxi Peng, Honggen Yuan, Zhanjie Jiang, Xiaoqing Ou, Qian Zhang, Kexin Yi, Yanbin Meng, Qun Xie

**Affiliations:** 1 School of Public Health, Xiangnan University, Chenzhou, 423000, China; 2 Department of Basic Medicine, Xiangnan University, Chenzhou, 423000, China; 3 Teaching and Research Section of Surgery, Xiangnan University Affiliated Hospital, Chenzhou, 423000, China

**Keywords:** N6-methyladenosine (m^6^A), c-Src, interferon regulatory factor 1 (IRF1), liver cancer, hepatocellular carcinoma (HCC)

## Abstract

**Background:**

Expression of mRNA is widely regulated by N6-methyladenosine (m^6^A). An increasing number of studies have shown that m^6^A methylation, facilitated by methyltransferase 3 (METTL3), is crucial in the progression of tumors. Previous reports have indicated the involvement of both METTL3 and c-Src kinase in the evolution of liver cancer. However, the potential connection between c-Src and the METTL3-mediated mechanism in liver cancer progression remains elusive.

**Methods:**

The correlation expression between c-Src and METTL3 between liver cancer patients and the control group was analyzed using the TCGA database, and was further demonstrated by Western blot and RT-qPCR. The functional roles of c-Src in METTL3-regulated liver cancer progression were investigated by cell proliferation assays and colony formation assays. The regulatory mechanism of METTL3 in c-Src expression was accessed by RNA-immunoprecipitation (RIP)-qPCR.

**Results:**

We demonstrated that c-Src kinase promoted liver cancer development, and the expression of *SRC* (encodes c-Src kinase) was positively correlated with METTL3 in liver cancer cases. We showed that *SRC* mRNA could be m^6^A-modified, and METTL3 regulated the transcription of *SRC* mRNA through interferon regulatory factor 1 (IRF1). We revealed that IRF1, the expression of which was positively regulated by METTL3, was a novel transcription factor of c-Src. Lastly, The pro-proliferative effect of METTL3 on hepatocellular carcinoma was mechanistically linked to IRF1/c-Src axis activation, as evidenced by our experimental data.

**Conclusion:**

Results suggested that the METTL3/IRF1/c-Src axis played potential oncogenic roles in liver cancer development and the axis may be a promising therapeutic target in the disease.

## 1 Introduction

Primary liver cancer ranks as the sixth most commonly diagnosed cancer, there were over three quarters of a million liver cancer deaths worldwide in 2022, positioning liver cancer as the third leading cause of cancer death after lung and colorectum, with an estimated 865,000 new cases and 757,948 deaths in 2022 [1]. Hepatocellular carcinoma (HCC), as the most common histological subtype of primary liver cancer, constitutes 75% to 85% of all cases globally, making it the predominant form of hepatic malignancies [2]. With the reduction of hepatitis virus-induced liver cancer [3], nonviral risk factors [4] have increasingly garnered attention in liver cancer research [5]. Essentially, identifying novel targets that drive the progression of liver cancer is necessary for the development of targeted therapy against this deadly disease.

N6-methyladenosine (m^6^A) represents the most prevalent internal modification in eukaryotic mRNAs [6]. m^6^A methylation is catalyzed by “writer” methyltransferases 3 (METTL3) and methyltransferase 14 (METTL14) and is demethylated by “eraser” demethylases AlkB homolog 5 (ALKBH5) and obesity-associated protein (FTO) and fat mass [7]. The coordinated interplay between m^6^A methyltransferases and demethylases orchestrates the spatiotemporal precision of RNA methylation homeostasis in mammalian cells, forming a dynamic reversible modification network critical for gene expression regulation. m^6^A modification functions as a crucial regulator of the mRNA life cycle, including pre-mRNA splicing [8], nucleo-cytoplasmic export [9], mRNA decay [10], and mRNA translation [11], and thereby regulating various cellular biological processes.

A growing body of work provides evidence that m^6^A promotes the development of cancers [12]. As the executor of m^6^A modification, METTL3 plays a crucial role in tumorigenesis. For instance, METTL3 is essential for the development and maintenance of myeloid leukemia in both mice and humans [13], modulates the Wnt/β-catenin-EMT axis in ESCC by stabilizing β-catenin/TCF4 transcriptional complexes to facilitate tumor invasion [14], and facilitates TGFβ signals to support tumor growth [15]. In HCC, METTL3-mediated m^6^A modification stabilizes ASPM mRNA via YTHDF1 recognition, thereby augmenting HCC cell proliferative capacityand metastatic potential [16]. Moreover, METTL3 is abnormally upregulated in HCC, and its expression has been shown to predict poor survival outcomes in HCC patients [17,18].

Classified as a non-receptor tyrosine kinase within the Src family kinases (SFKs), c-Src orchestrates key cellular dynamics including differentiation, motility, and intercellular adhesion through phosphorylation signaling [19]. C-Src kinase can be activated by diverse stimuli, including epidermal growth factor receptor (EGFR) [20], P2RY2 (a purinergic GPCR receptor), reactive oxygen species (ROS) [21], high glucose [22], heterotrimeric G protein-coupled receptors [23], and PKA signaling [24]. Increasing evidence shows that c-Src is a critical factor in the development of a variety of human cancers [25], such as liver cancer [26–28]. However, it remains unclear if c-Src is associated with the METTL3-regulated mechanism in liver cancer.

In this study, we bridged the association between *SRC* and *METTL3* according to publicly available liver cancer datasets with clinical data and functional analyses revealed that METTL3 modulates SRC mRNA expression through m^6^A-dependent mechanisms. We further provided evidence showing that IRF1 was a novel transcription factor of SRC, and the IRF1/c-Src axis participated in METTL3-promoted proliferation of liver cancer cells.

## 2 Materials and Methods

### 2.1 Cell Culture, Treatments, and Transfection

Liver cancer cell lines Huh7 and HepG2 were used for investigation in this study. Stably METTL3 silencing Huh7 and cells (sh-*METTL3* Huh7), its control cells (sh-NC Huh7) were the gift from Prof. Hongsheng Wang (School of Pharmaceutical Sciences, Sun Yat-Sen University). Use validated siRNA sequences for METTL3 (5^′^-GCCAAGGAACAATCCATTGTT-3^′^), silencing METTL3, transfected into HepG2 cells. IRF1 cDNA cloned into an expression vector (pcDNA3.1), transfected into sh-*METTL3* Huh7 or si*METTL3* HepG2 cells. Use validated siRNA sequences for c-Src(5^′^-AAGUGCAAUGUGGACCGUAUC-3^′^) and IRF1(5^′^-CUGAAGAGCUUCAGCAUCA-3^′^), silencing either c-Src or IRF1, transfected into Huh7 or HepG2 cells. Cells were cultured in Dulbecco’s Modified Eagle Medium (DMEM) (Gibco, 11965118, Carlsbad, CA, USA) supplemented with 10% fetal bovine serum (FBS) (Gibco, 26140111, Carlsbad, CA, USA) and 0.5 μg/mL Mycoplasma removal reagent (Beyotime, C0288S, Shanghai, China) at 37°C in a 5% CO_2_ incubator. For transfection, plasmids were transfected into cells using Lipofectamine^TM^ 3000 reagent (Invitrogen Life Technology, L3000001, Carlsbad, CA, USA) following the manufacturer’s instructions.

### 2.2 Plasmid Constructions

The promoter region of *SRC* (encodes for c-Src kinase) was cloned into the pGL3 basic vector upstream of the F-Luc gene. Mammalian expression plasmids pcDNA3-*METTL3* and pcDNA3-*METTL3*-D395A were gifts from Prof. Hongsheng Wang (School of Pharmaceutical Sciences, Sun Yat-Sen University, Guangzhou, China).

### 2.3 Western Blot Analysis

Cells were harvested and lysed in RIPA buffer (50 mM Tris-HCl pH 7.4, 150 mM NaCl, 1% Triton X-100, 1% sodium deoxycholate, 0.1% SDS, 0.5 mM PMSF). Total proteins were extracted and quantified by BCA assay (Thermo, Prod# 23228), and 5× loading buffer was added to the proteins. A total of 30 μg of proteins from each sample were loaded and separated in 12% SDS-PAGE, then transferred onto polyvinylidene fluoride (PVDF) membranes (ThermoFisher Scientific, LC2005, Waltham, MA, USA), and 5% defatted milk powder dissolved in TBST (20 mM Tris, pH 7.4, 150 mM NaCl, 0.1% Tween 20) was used for blocking. PVDF membrane was incubated with primary antibodies overnight at 4°C. After three times TBST washing, membranes were incubated with secondary antibodies for 1 h at room temperature. After washing with TBST, proteins in PVDF membrane bands were detected with luminol reagent (Santa Cruz, SC-2385, Dallas, TX, USA). GAPDH expression was used as the housekeeping protein. The primary antibodies used for immunoblotting included anti-c-Src (Abcam, ab109381, Cambridge, UK), anti-METTL3 (Abcam, ab195352, Cambridge, UK), anti-IRF1 (Abcam, ab245338, Cambridge, UK), and anti-GAPDH (Cell Signaling Technology, 5174S, Danvers, MA, USA) with 1:1000 dilution. The goat anti-rabbit IgG H&L (HRP) (Abcam, ab205718, Cambridge, UK) was used as secondary antibodies, with 1:5000 dilution. The immunoblotting results were representatives from at least three independent experiments. The band intensity of detected proteins was analyzed by ImageJ software (ImageJ 1.50i, National Institutes of Health, Bethesda, MD, USA), and the presented values below each protein band were the mean from three independent experiments.

### 2.4 RNA Extraction and Quantitative Real-Time PCR (qRT-PCR)

Total RNA was extracted from cells using Trizol reagent (TaKaRa, 9108Q, Beijing, China), and 1 μg total RNA was used as the template for cDNA synthesis in PrimeScript^®^ First Strand cDNA Synthesis kit (TaKaRa, D6110A, Beijing, China). qRT-PCR was performed in the SYBR^®^ Premix Ex Taq^TM^ kit (TaKaRa, RR390A, Beijing, China) using specific primers. *GAPDH* was used as a control for normalization. The relation expression of tested genes was calculated by the cycle threshold values (Ct) as 2^−ΔΔCt^, where ΔCt = Gene Ct-Housekeeping gene Ct, ΔΔCt = ΔCt-Reference ΔCt, and Reference ΔCt = average of control group Ct. The primers of targeted human genes were as follows: *SRC*, forward 5^′^-AAGCCTGGCACGATGTCT-3^′^ and reverse 5^′^-CGATGTAAATGGGCTCCTCT-3^′^; pre-mRNA *SRC*, forward 5^′^-ATCTCATTGTGGTTTTGATT-3^′^ and reverse 5^′^-TGCGAGGATCACTTGAGCCC-3^′^; *IRF1*, forward 5^′^-AGAGAAAAGAAAGAAAGTCG-3^′^ and reverse 5^′^-TGGGCTGTCAATTTCTGGCT-3^′^; *SRC* promoter, forward 5^′^-TTCTTGTCAGTGCCTCAGTT-3^′^ and reverse 5^′^-CTTCTACGCCCCAGATCCGC-3^′^; *GAPDH*, forward 5^′^-GTCTCCTCTGACTTCAACAGCG-3^′^ and reverse 5^′^-ACCACCCTGTTGCTGTAGCCAA-3^′^; *METTL3*, forward 5^′^-CTATCTCCTGGCACTCGCAAGA-3^′^ and reverse 5^′^-GCTTGAACCGTGCAACCACATC-3^′^; *FLUC*, forward 5^′^-GGCCTGACAGAAACAACCAG-3^′^ and reverse 5^′^-AAGTCCACCACCTTAGCCTC-3^′^; *RLUC*, forward 5^′^-CGCTATTGTCGAGGGAGCTA-3^′^ and reverse 5^′^-GCTCCACGAAGCTCTTGATG-3^′^; *HPRT1*, forward 5^′^-TGACACTGGCAAAACAATGCA-3^′^ and reverse 5^′^-GGTCCTTTTCACCAGCAAGCT-3^′^; *18S*, forward 5^′^-CGGACAGGATTGACAGATTGATAGC-3^′^ and reverse 5^′^-TGCCAGAGTCTCGTTCGTTATCG-3^′^.

### 2.5 m^**6**^A RNA-Immunoprecipitation (RIP) qPCR

The m^6^A-qRT-PCR was conducted according to published protocols [15]. A total of 200 µg total RNAs extracted by Trizol were used for immunoprecipitation by the m^6^A antibody (Abcam, ab286164, Cambridge, UK) or IgG (Abcam, ab172730, Cambridge, UK) in IP buffer (150 mM NaCl, 0.1% NP-40, 10 mM Tris, pH 7.4, 100U RNase inhibitor). The m^6^A RNAs were immunoprecipitated by Dynabeads® Protein G (ThermoFisher Scientific, 10003D, Waltham, MA, USA) and eluted twice by elution buffer (5 mM Tris-HCl pH 7.5, 1 mM EDTA pH 8.0, 0.05% SDS, 20 mg/mL Proteinase K). RNAs were then precipitated by ethanol and the RNA concentration was measured with a Qubit® RNA HS Assay Kit (ThermoFisher Scientific, Q32852, Waltham, MA, USA). A total of 2 ng of total RNA (input) and m^6^A-IP RNA were used as the template for qRT-PCR. Hypoxanthine phosphoribosyl transferase 1 (HPRT1) was used as the internal control of the input samples.

### 2.6 mRNA Stability Assay

Actinomycin D (Act-D; 5 μg/mL) (Sigma, SBR00013, Taufkirchen, Germany) was added to the serum-free culture medium for the indicated times. The cells were then washed by PBS and subjected to total RNA extraction by Trizol. RNA concentrations were quantified and qRT-PCR was performed. Detection of *SRC* mRNA levels represented the stability. *18S* mRNA was used as the internal control.

### 2.7 Cell Fractionation Assay

Fractionation of the nuclear and cytoplasmic samples was performed using an NE-PER(R) nuclear and cytoplasmic extraction kit (ThermoFisher Scientific, 78833, Waltham, MA, USA). Total RNAs in the nuclear and cytoplasmic fractions were extracted by Trizol. The nuclei–cytoplasm ratio was determined by the mRNA levels of targets in the nuclear and cytoplasmic fractions, which were normalized to the levels of nuclear Metastasis Associated Lung Adenocarcinoma Transcript 1 (*MALAT1*) RNA and cytoplasmic *7SL* RNA, respectively.

### 2.8 Dual-Luciferase Reporter Assay

The luciferase assay was performed according to the manufacturer’s instructions (Beyotime Biotechnology, Beijing, China). Briefly, cells were co-transfected with pGL3–basic derived by *SRC* promoter and TK-R-luc reporter in a 6-well plate for 48 h. Cells were then analyzed with the Dual-Glo Luciferase Assay system (Promega, E2920, Madison, WI, USA). Renilla luciferase (RLUC) was used to normalize firefly luciferase (FLUC) activity.

### 2.9 Chromatin Immunoprecipitation (ChIP)-qPCR

ChIP was performed using the Magna ChIP™ A/G Chromatin Immunoprecipitation Kit (Merck Millipore, 17-10085, Burlington, MA, USA) with an antibody specific for IRF1 (Abcam, ab243895, Cambridge, MA, USA; 2 µL for 10^7^ cells) or normal rabbit IgG (Santa Cruz Biotechnology, sc-2357, Santa Cruz, CA, USA; 2 µL of 1:10 dilution). Following ChIP, quantitative PCR was utilized to amplify and quantify the immunoprecipitated DNA using primers specific for the IRF1 binding region on the *SRC* promoter. The IRF1 binding site within the *SRC* promoter region was obtained from the ENCODE Consortium. The ChIP-qPCR values were normalized to that of input control and represented as fold enrichment relative to the anti-normal rabbit IgG control group, using *HPRT1* as internal control.

### 2.10 Cell Proliferation Assay

Cell proliferation was evaluated by CCK8 kit (Abcam, ab228554, Cambridge, UK) assay at indicated time points. 10^6^ cells were seeded in 6-well plates one day before treatment. Cells were transfected with or without pcDNA3.1-*SRC* and control vectors. After transfection for 24 h, cells were trypsinized and seeded in 96-well plates (6 × 10^3^ cells/well). CCK8 reagent was added in cells for a further 2 h incubation at 37°C. Subsequently, the OD values at 450 nm were recorded, and the cell proliferation diagram was plotted using the absorbance at each time point.

### 2.11 Colony Formation Assay

Cells were seeded in 6-well plates (1 × 10^3^ cells/well) one day before treatment. The next day, cells were transfected with si-*SRC*, pcDNA3.1-*SRC*, or pcDNA3-*METTL3* and control vectors as indicated. After transfection for 24 h, cells were trypsinized and re-suspended. A total of 1000 cells/well were seeded in 6-well plates, and cultured for 14 days. Cell colonies were gently washed by PBS, and then fixed by 4% paraformaldehyde for 30 min. After PBS washing, cell colonies were incubated with crystal violet (1 mL crystal violet stock solution, add 9 mL PBS buffer solution) for 20 min. Photo recording was performed after PBS washing.

### 2.12 Bioinformatic Analysis

Expression levels of interested genes in tumor and normal tissues from liver cancer patients, survival analysis, and correlation analysis were performed using the online database Gene Expression Profiling Interactive Analysis 2 (GEPIA2) http://gepia2.cancer-pku.cn/#index (accessed on 20 December 2024).

### 2.13 Statistical Analysis

For the comparison of numerical variables between two groups, an unpaired *t*-test was used for normally distributed variables. For correlation analysis between two sets of numerical variables, Pearson correlation coefficient was used. Student’s *t*-test was used for the comparison of the mean equality of two independent samples, including the mRNA stability assay. One-way ANOVA analysis was used for the comparison of multiple groups. All analysis was conducted using GraphPad Prism version 9.0 (GraphPad Inc., La Jolla, CA, USA) and two-tailed *p* < 0.05 was considered statistically significant.

## 3 Results

### 3.1 Both SRC and METTL3 Are Associated with Worse Survival in Liver Cancer Patients

**Figure 1:**
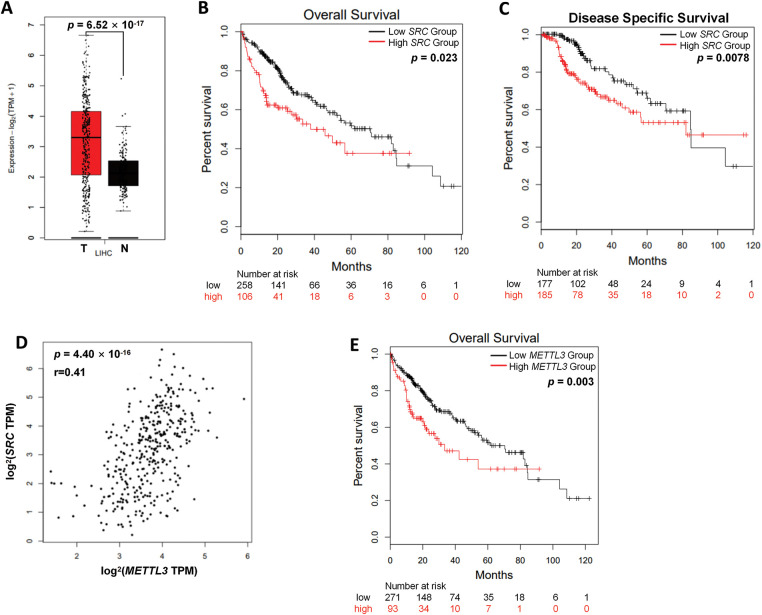
Both *SRC* and *METTL3* are associated with worse prognosis in liver cancer patients. (**A**) The relative mRNA expression of *SRC* in liver cancer patients (*T*, *n* = 369) compared with normal controls (*N*, *n* = 160) from TCGA datasets; (**B, C**) the Kaplan–Meier survival curves of overall survival (**B**) and disease-free survival (**C**) based on *SRC* expression in liver cancer patients; (**D**) association between *SRC* and *METTL3* in liver cancer patients; (**E**) the Kaplan–Meier survival curves of overall survival based on *METTL3* expression in liver cancer patients. The group cutoff for analysis in (**B, C, E**) was 50%

To analyze the potential role of c-Src in liver cancer, we first examined the *SRC* (code for c-Src) mRNA expression levels in the tumor and normal tissues from liver cancer patients of TCGA series of cases based on Gene Expression Profiling Interactive Analysis 2 (GEPIA2) database [29]. Hepatocellular carcinoma samples displayed a dramatic surge in SRC expression compared with non-cancerous hepatic controls. (*p* = 6.52 × 10^−17^) (Fig. 1A). Kaplan-Meier analysis revealed that liver patients with increased *SRC* expression demonstrated significantly poorer overall survival (OS) (*p* = 0.023) (Fig. 1B) and disease-specific survival (*p* = 7.80 × 10^−3^) (Fig. 1C). Results confirmed the oncogenic role of c-Src in liver cancer.

Next, the potential linkage between *SRC* and *METTL3* in liver cancer was investigated. According to GEPIA2 analysis, there was a significant positive correlation between *SRC* and *METTL3* expression in liver cancer tissues was observed (r = 0.41; *p* = 4.40 × 10^−16^) (Fig. 1D). Furthermore, liver cancer patients with higher *METTL3* expression showed poorer OS (*p* = 3.00 × 10^−3^) (Fig. 1E). Together, results showed that both *SRC* and *METTL3* were associated with worse survival of liver cancer patients.

### 3.2 METTL3 Regulates the Expression of c-Src in Liver Cancer Cells

**Figure 2:**
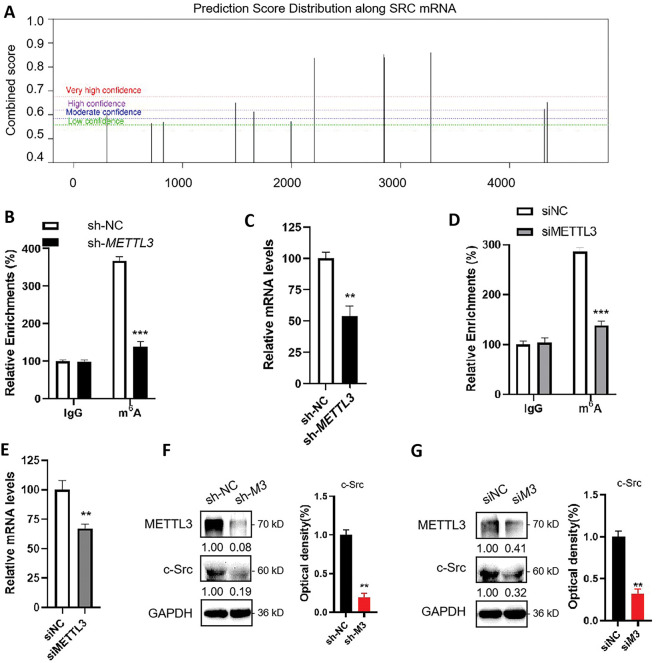
METTL3 regulates the expression of c-Src in liver cancer cells. (**A**) Prediction of m^6^A modifications on *SRC* mRNA; (**B**) m^6^A RIP-qPCR analysis of *SRC* mRNA in sh-NC and sh-*METTL3* Huh7 cells. Enrichment of *SRC* mRNA in m^6^A RIP samples was normalized to IgG and sample input; (**C**) Expression of *SRC* mRNA in sh-NC and sh-*METTL3* Huh7 cells; (**D**) m^6^A RIP-qPCR analysis of *SRC* mRNA in siNC and si*METTL3* HepG2 cells. Enrichment of *SRC* mRNA in m^6^A RIP samples was normalized to IgG and sample input; (**E**) Expression of *SRC* mRNA in siNC and si*METTL3* HepG2 cells; (**F**) Expression of METTL3 and c-Src in sh-NC and sh-*METTL3* (sh-M3) in Huh7 cells; (**G**) Expression of METTL3 and c-Src in siNC and si*METTL3* (siM3) HepG2 cells. Data of (B–G) are presented as means ± SD from three independent experiments. Student’s *t*-test, **p < 0.01; ***p < 0.001.

Since METTL3 is one of the key regulators of m^6^A, which commonly participates in the regulation of mRNA expression, results hinted that METTL3 may regulate the expression of *SRC*. We therefore hypothesized that *SRC* mRNA was a target of m^6^A modification. Firstly, we examined for possible m^6^A modification sites on *SRC* mRNA using the online tool SRAMP (http://www.cuilab.cn/sramp/, accessed on 20 December 2024). Results showed that there were three predicted m^6^A sites with very high confidence (Fig. 2A). Subsequently, we performed m^6^A-RIP-qPCR using control (sh-NC) Huh7 cells and *METTL3* knockdown (sh-*METTL3*) Huh7 cells (Fig. 2B). RT-qPCR showed significant enrichment of *SRC* mRNA in the m^6^A-IP group in sh-NC Huh7 cells (Fig. 2C), indicating the presence of m^6^A modification on *SRC* mRNA. In sh-*METTL3* Huh7 cells, *SRC* mRNA was no longer enriched in the m^6^A-IP group (Fig. 2B), suggesting the m^6^A modification on *SRC* mRNA was reversible. Similar observations were obtained when using HepG2 cells with siNC or si*METTL3* knockdown (Fig. 2D,E). Together, it suggested that SRC mRNA can be m^6^A modified reversely.

In line with that mRNA expression is commonly regulated by m^6^A modifications, RT-qPCR results showed a significant reduction of *SRC* mRNA expression in sh-*METTL3* Huh7 cells and siMETTL3 HepG2 cells (Fig. 2C,E). This result was consistent with the clinical data presented in Fig. 1E. Consistently, c-Src protein levels were significantly decreased in sh-*METTL3* Huh7 cells and siMETTL3 HepG2 cells (Fig. 2F,G). Together, these data suggested that *SRC* mRNA can be m^6^A modified via METTL3, and METTL3 upregulated the expression of c-Src in liver cancer cells.

### 3.3 m^**6**^A Modulates the Transcription of SRC mRNA in Liver Cancer Cells

**Figure 3:**
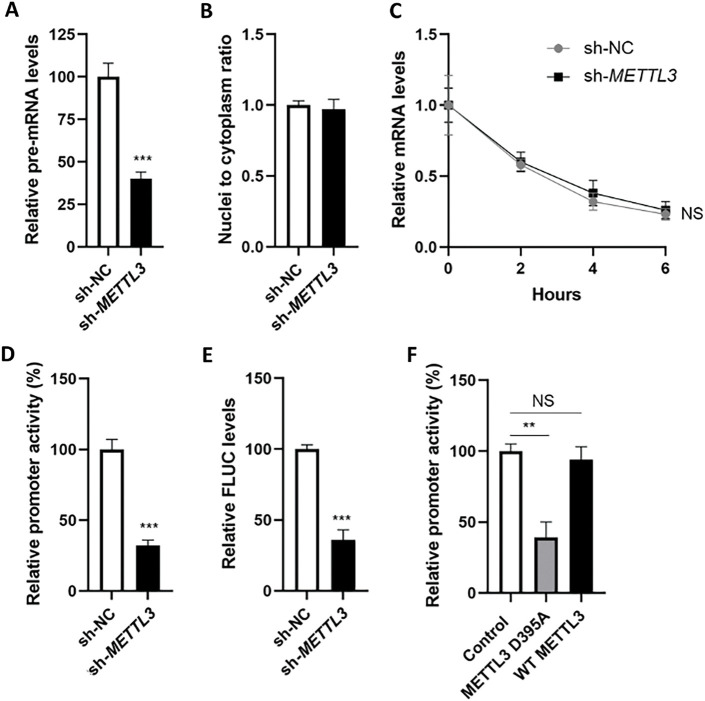
m^6^A modulates the transcription of *SRC* mRNA in liver cancer cells. (**A**) Expression of *SRC* precursor mRNA in sh-NC and sh-*METTL3* Huh7 cells; (**B)** ratio of nuclei-to-cytoplasm *SRC* mRNA in sh-NC and sh-*METTL3* Huh7 cells; (**C**) half-lives of *SRC* mRNA in sh-NC and sh-*METTL3* Huh7 cells; (**D**) relative promoter activity of dual F-Luc reporter derived by *SRC* promoter in sh-NC and sh-*METTL3* Huh7 cells; (**E**) expression of *FLUC* mRNA from dual F-Luc reporter derived by *SRC* promoter in sh-NC and sh-*METTL3* Huh7 cells; (**F**) relative promoter activity of dual F-Luc reporter derived by *SRC* promoter in sh-*METTL3* Huh7 cells overexpressing wild type METTL3 (WT METTL3) or catalytic mutant (METTL3 D395A). Data are presented as means ± SD from three independent experiments. Student’s *t*-test (**A, B, D–F**) or One-way ANOVA (**C**), **p < 0.01; ***p < 0.001 compared with control; ns no significance

m^6^A modification is associated with almost all biological processes of mRNA, including transcription, nucleic-cytoplasm transport, and mRNA decay. RT-qPCR results showed that the pre-mRNA level of *SRC* was significantly decreased in sh-*METTL3* Huh7 cells (Fig. 3A). However, neither the nuclear-to-cytoplasm ratio of *SRC* mRNA (Fig. 3B) nor the haft-life of *SRC* mRNA (Fig. 3C) showed a significant difference between sh-NC Huh7 and sh-*METTL3* Huh7 cells. Taken together, the results suggested that METTL3 may affect the pre-mRNA level of *SRC*.

To verify whether METTL3 regulated the transcription of *SRC*, we constructed a dual-luciferase reporter consisting of *SRC* promoter. Results from dual-luciferase assays showed that fluorescent signals (F-Luc) were suppressed in sh-*METTL3* Huh7 cells (Fig. 3D). Consistently, the mRNA level of firefly luciferase (*FLUC*) was significantly decreased in sh-*METTL3* Huh7 cells (Fig. 3E). After overexpressing wild-type or non-catalytic METTL3 in sh-*METTL3* Huh7 cells, F-Luc products were recruited in cells overexpressing wild-type METTL3, but not the non-catalytic METTL3 (Fig. 3F). Together, results indicated that METTL3 modulated the transcription of *SRC* in liver cancer cells.

### 3.4 IRF1 Is a Novel Transcription Factor of c-Src That Is Regulated by METTL3

**Figure 4:**
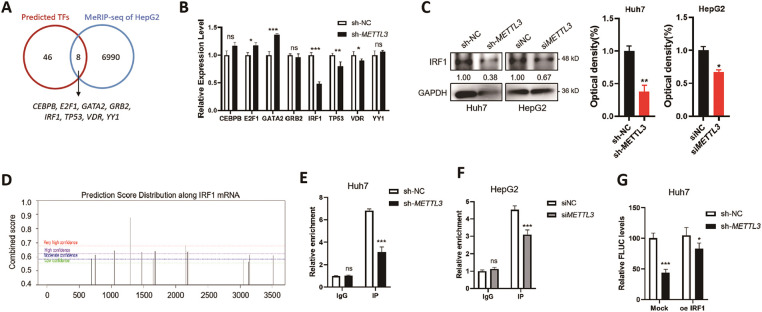

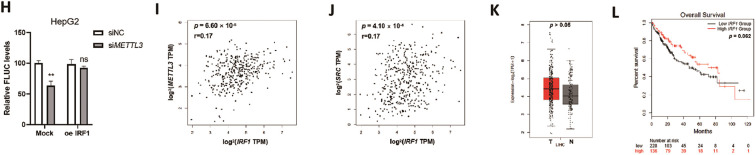
IRF1 is a novel transcription factor of c-Src that is regulated by METTL3. (**A**) Overlapping of online predicted TFs of *SRC* and the m^6^A-modified genes in HepG2 cells from MeRIP-sequencing dataset (GSE37003); (**B**) relative expression levels of m^6^A-modified predicted TFs of *SRC* in sh-NC and sh-*METTL3* Huh7 cells; (**C**) expression of IRF1 in sh-NC and sh-*METTL3* Huh7 cells, and siNC and siMETTL3 HepG2 cells; (**D**) prediction of m^6^A modifications on *SRC* mRNA; (**E, F**) m^6^A RIP-qPCR analysis of *IRF1* mRNA in sh-NC and sh-*METTL3* Huh7 cells (**E**) and siNC and siMETTL3 HepG2 cells (**F**). Enrichment of *IRF1* mRNA in m^6^A RIP samples was normalized to IgG and sample input; (**G, H**) expression of *FLUC* mRNA from dual F-Luc reporter derived by *SRC* promoter in sh-NC and sh-*METTL3* Huh7 cells (**G**) and siNC and siMETTL3 HepG2 cells (**H**) with or without IRF1 overexpression; (**I**) correlation between *METTL3* and *IRF1* in liver cancer patients from TCGA datasets; (**J**) correlation between *SRC* and *IRF1* in liver cancer patients from TCGA datasets; (**K**) the relative mRNA expression of *IRF1* in liver cancer patients (*T*, *n* = 369) compared with normal controls (*N*, *n* = 160) from TCGA datasets; (**L**) the Kaplan-Meier survival curves of overall survival based on *IRF1* expression in liver cancer patients. Data of (**B, E–H**) are presented as means ± SD from three independent experiments. Student’s *t*-test, *p < 0.05; **p < 0.01; ***p < 0.001; ns, no significant compared with controls

To explore how m^6^A regulates the transcription of c-Src, potential transcription factors (TFs) of c-Src were predicted via the JASPAR online tool (https://jaspar.elixir.no/, accessed on 20 December 2024). Results showed that there were 46 predicted TFs for c-Src, and 8 of them (*CEBPB*, *E2F1*, *GATA2*, *GRB2*, *IRF1*, *TP53*, *VDR*, and *YY1*) were identified as m^6^A targets (GSE37003; Fig. 4A). We assumed that the expressions of potential TFs for c-Src might be modulated by m^6^A and therefore affect the transcription of c-Src. RT-qPCR results of 8 TF candidates were examined in sh-NC and sh-*METTL3* Huh7 cells. Results showed that *IRF1* mRNA was significantly downregulated in sh-*METTL3* Huh7 cells (Fig. 4B). Similarly, Western blot analysis showed a decrease of IRF1 in sh-*METTL3* Huh7 and si-*METTL3* HepG2 cells (Fig. 4C). The reduction of IRF1 in METTL3 knockdown or silencing cells may be due to the positive regulation of m^6^A modification of IRF1 mRNA since there was one predicted m^6^A site with high confidence (Fig. 4D). However, the regulation of m^6^A of IRF1 mRNA expression remained to be explored. Together, results indicated that IRF1 might be a potential TF of *SRC*, which was associated with the m^6^A-regulated *SRC* transcription.

By using ChIP-qPCR, it was confirmed that IRF1 can bind to the promoter region of *SRC* in both Huh7 and HepG2 cells (Fig. 4E,F). In either sh-*METTL3* Huh7 or si*METTL3* HepG2 cells, the binding affinity between IRF1 and *SRC* promoter region significantly decreased (Fig. 4E,F), suggesting that IRF1 may promote the transcription of *SRC*. To further verify the transcriptional regulation of IRF1 on *SRC*, dual-luciferase assays were performed in either sh-*METTL3* Huh7 or si*METTL3* HepG2 cells overexpressing IRF1. Results showed that recruitment of IRF1 significantly enhanced the F-Luc levels (Fig. 4G,H). In addition, the expression of IRF1 was positively correlated to both *SRC* and *METTL3* in liver cancer patients, *p* = 6.60 × 10^−5^ (Fig. 4I) and *p* = 4.10 × 10^−4^ (Fig. 4J), respectively. However, the expression of IRF1 showed a non-significant difference between liver cancer tissues compared to the control group (Fig. 4K). In addition, IRF1 level did not statistically affect the OS (*p* = 0.062) of liver cancer patients (Fig. 4L), which might be due to the comprehensive effects of IRF1 in cells. Together, results suggested that IRF1 is a novel TF of *SRC* that is regulated by METTL3.

### 3.5 IRF1/c-Src Axis Is Associated with METTL3-Promoted Proliferation of Liver Cancer Cells

**Figure 5:**
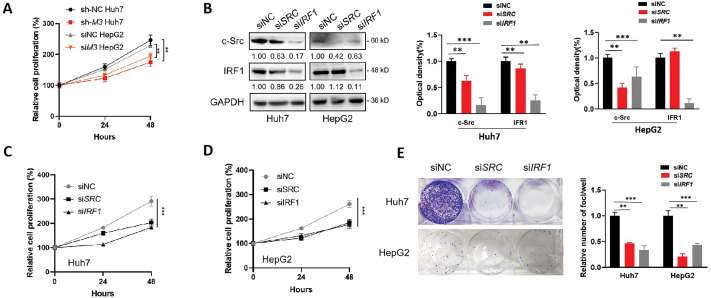

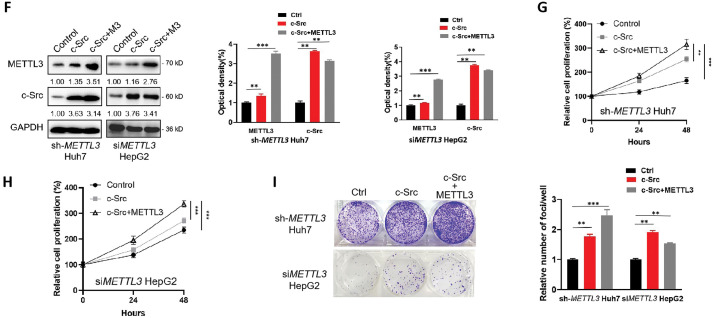
IRF1/c-Src is associated with the METTL3-promoted proliferation of liver cancer cells. (**A**) Relative cell proliferation of sh-NC and sh-*METTL3* Huh7 cells, and siNC and si*METTL3* HepG2 cells; (**B–E**) expression of c-Src and IRF1 (**B**), relative cell proliferation (**C, D**) and colony formation (**E**) of Huh7 or HepG2 cells silencing c-Src or IRF1; (**F–I**) Expression of c-Src and METTL3 (**F**), relative cell proliferation (**G, H**) and colony formation (**I**) of sh-*METTL3* Huh7 or si-*METTL3* HepG2 cells overexpressing c-Src with or without METTL3. Data of (**A, C, D, G, H**) are presented as means ± SD from three independent experiments. One-way *ANOVA*, **p < 0.01; ***p < 0.001 compared with controls

We hypothesized that the IRF1/c-Src axis was associated with the m^6^A-regulated development of liver cancer. In line with the oncogenic roles of METTL3, the knockdown of METTL3 in both Huh7 and HepG2 cells significantly suppressed their proliferation (Fig. 5A). When transiently silencing either c-Src or IRF1 expression (Fig. 5B), significant reductions in cell proliferation were observed (Fig. 5C,D). Notably, a more obvious reducing effect was obtained in cells silencing IRF1, which may be due to a more severe decrease of c-Src level (Fig. 5B) or other regulatory effects of IRF1. Consistently, similar results were obtained in colony formation assays using different liver cancer cells with silenced c-Src or IRF1 expression (Fig. 5E).

Since IRF1 unaffected the OS of liver cancer patients (Fig. 4L) and c-Src acts as an effector in the METTL3/IRF1/c-Src axis, we further overexpressed c-Src in sh-METTL3 Huh7 and siMETTL2 HepG2 cells, respectively, to confirm the oncogenic role of METTL3/IRF1/c-Src axis in liver cancer cell proliferation. After overexpressing c-Src, both proliferation and colony-forming capabilities of sh-*METTL3* Huh7 cells and siMETTL2 HepG2 cells were partially restored (Fig. 5F–I). Together, our results showed a critical role of the IRF1/c-Src axis in the METTL3-promoted liver cancer progression.

## 4 Discussion

Increasing evidence has shown that m^6^A methylation is associated with various biological functions and the onset of tumorigenesis [30,31]. Here, we reported that METTL3, acting as m^6^A methyltransferase, positively modulated the expression of c-Src kinase. In particular, METTL3 promoted the transcription of *SRC* via modulation of IRF1 expression, which was identified as a novel transfection factor of *SRC*. Last, we proved that the upregulation of IRF1/c-Src expression participated in the METTL3-promoted cell proliferation of liver cancer.

Our results indicated that METTL3 enhanced SRC transcription through a mechanism reliant on IRF1. The regulation of m^6^A on gene transcription is mediated by two major mechanisms: m^6^A directly regulation by affecting the chromatin accessibility and m^6^A mediates indirect regulation by altering the transcriptional activity of TFs linked to target genes. Recently, Liu et al. reported that knockout of METTL3 increased chromatin accessibility and activated transcription in a m^6^A-dependent manner [32]. Notably, regulation of m^6^A modifications on gene transcription is indirect as DNA does not contain m^6^A modification. For instance, the effects of m^6^A modifications stabilize chromosome-associated regulatory RNAs (carRNAs) and therefore suppress transcription [32]. Another mechanism for the m^6^A-regulated transcription process might be related to the expression changes of transcription factors. This is exemplified by reports that m^6^A can modulate the expression of bromodomain PHD finger transcription factor (BPTF), leading to the upregulation of *SRC* transcription [33]. Here, we reported that IRF1 was a novel TF of *SRC*, which can positively enhance the *SRC* transcription. Furthermore, we found that *IRF1* was also a potential m^6^A target like SRC, which was confirmed by MeRIP-qPCR and online prediction. However, whether m^6^A affected the binding between IRF1 and *SRC* promoter, and how m^6^A regulated the expression of IRF1 remained further explored.

The relationship between m^6^A and c-Src appears to be m^6^A catalyst type-dependent and/or cancer type-dependent. For instance, the m^6^A methylase METTL14 was reported to negatively regulate the expression of *SRC* in renal cell carcinoma [33]. In contrast, the EGFR/SRC/ERK signaling pathway can phosphorylate and stabilize the m^6^A reader protein YTHDF2, which is necessary to sustain the invasive, proliferative, and tumorigenic properties of glioblastomas [34]. In liver cancer, the relationship between METTL3 and c-Src has seldom been reported. Our findings revealed that METTL3 upregulates SRC expression in hepatocellular carcinoma through IRF1-dependent regulation. Clinical data analysis showed a positive correlation between both METTL3/SRC and METTL3/IRF1, as well as the OS of liver cancer patients associated with METTL3/SRC expression. These data hinted at the potential roles of the METTL3/IRF1/c-Src axis in the development of liver cancer. Notably, the METTL3-regulated *SRC* expression might be cancer type-specific and the relationship between METTL3 and c-Src in other types of cancer remains an open question.

Modulating m^6^A modification has emerged as a pivotal focus in oncological therapeutic research over the past decade. One of the common strategies is inhibiting global m^6^A levels, such as the usage of small molecule inhibitors targeting METTL3 (i.e., STM2457). STM2457 has been identified and characterized where the inhibitor highly and selectively inhibited METTL3 activities [35]. More importantly, STM2457 attenuated the growth of leukemia cells and promoted their differentiation as well as apoptosis *in vitro*, and the METTL3 inhibitor prolonged the survival of leukemia mice models by impairing leukemia cell engraftment and suppressing leukemic stem cell subpopulations. However, the anti-tumor effect of targeting m^6^A is not efficient enough in the treatment of solid cancers. Therefore, discovering novel targets and combining m^6^A inhibition might be an emergent direction in enhancing m^6^A-dependent anti-cancer therapy [36,37]. Herein, we revealed that both IRF1 and c-Src showed promoted effects on the proliferation of liver cancer cells, hinting that either of them might be the potential target of liver cancer therapy. However, the combined inhibition effect between IRF1/c-Src and METTL3 required investigation.

We acknowledge the limitations of the study as follows: (1) Lack of *in vivo* data to validate our *in vitro* findings; (2) The regulation of m^6^A on IRF1 expression remained to be explored; (3) It is unlikely that c-Src kinase acted as the sole factor that participated in METTL3-regulated liver cancer cell proliferation, due to METTL3 has been reported to modulate multiple factors in cells through its m^6^A modification activities. For example, Snail (an EMT-inducing transcription factor) and the growth factor TGFβ1 were previously reported to be regulated by METTL3 required for the development of cancers [15].

## 5 Conclusion

In summary, we showed that METTL3/IRF1/c-Src axis played oncogenic roles in the development of liver cancer. Furthermore, we demonstrated that METTL3 positively regulated the transcription of c-Src kinase via IRF1, which was a novel TF of *SRC*. We further confirmed that the IRF1/c-Src axis promoted the METTL3-regulated proliferation of liver cancer cells. Detailed mechanisms on the METLL3/IRF1/c-Src axis in liver tumorigenesis and their effects *in vivo* warrant further investigations.

## Data Availability

Not applicable.
